# Disturbed flow regulates protein disulfide isomerase A1 expression via microRNA-204

**DOI:** 10.3389/fphys.2024.1327794

**Published:** 2024-04-04

**Authors:** Leonardo Y. Tanaka, Sandeep Kumar, Lucas F. Gutierre, Celso Magnun, Daniela Kajihara, Dong-Won Kang, Francisco R. M. Laurindo, Hanjoong Jo

**Affiliations:** ^1^ Vascular Biology Laboratory, Heart Institute (InCor), University of São Paulo, School of Medicine, São Paulo, Brazil; ^2^ Wallace H. Coulter Department of Biomedical Engineering, Emory University and Georgia Institute of Technology, Atlanta, GA, United States

**Keywords:** protein disulfide isomerase, microRNA, vascular smooth muscle cell, disturbed flow, atherosclerosis, phenotype switch

## Abstract

Redox processes can modulate vascular pathophysiology. The endoplasmic reticulum redox chaperone protein disulfide isomerase A1 (PDIA1) is overexpressed during vascular proliferative diseases, regulating thrombus formation, endoplasmic reticulum stress adaptation, and structural remodeling. However, both protective and deleterious vascular effects have been reported for PDIA1, depending on the cell type and underlying vascular condition. Further understanding of this question is hampered by the poorly studied mechanisms underlying PDIA1 expression regulation. Here, we showed that PDIA1 mRNA and protein levels were upregulated (average 5-fold) in the intima and media/adventitia following partial carotid ligation (PCL). Our search identified that miR-204-5p and miR-211-5p (miR-204/211), two broadly conserved miRNAs, share PDIA1 as a potential target. MiR-204/211 was downregulated in vascular layers following PCL. In isolated endothelial cells, gain-of-function experiments of miR-204 with miR mimic decreased PDIA1 mRNA while having negligible effects on markers of endothelial activation/stress response. Similar effects were observed in vascular smooth muscle cells (VSMCs). Furthermore, PDIA1 downregulation by miR-204 decreased levels of the VSMC contractile differentiation markers. In addition, PDIA1 overexpression prevented VSMC dedifferentiation by miR-204. Collectively, we report a new mechanism for PDIA1 regulation through miR-204 and identify its relevance in a model of vascular disease playing a role in VSMC differentiation. This mechanism may be regulated in distinct stages of atherosclerosis and provide a potential therapeutic target.

## Introduction

Although redox processes are closely associated with the vascular system under physiological or pathological conditions, specific mechanisms are complex and need further investigation. Protein disulfide isomerase A1 (PDIA1), a dithiol/disulfide redox chaperone highly expressed in the endoplasmic reticulum, is reportedly upregulated in vascular diseases such as atherosclerosis in humans and in experimental models of atherosclerotic disease (Tanaka et al., 2016). However, the role of PDIA1 has been unclear due to conflicting reports in vascular disease development. Total PDIA1 levels positively correlate with preserved endothelial function (Kim et al., 2018). In contrast, however, PDIA1 expression is enriched in vascular wall areas with marked cell death and proliferation in experimental atherosclerosis (Ping et al., 2017) and also correlates with plaque instability in human atherosclerosis (Tanaka et al., 2016). Moreover, PDIA1 levels are upregulated under conditions of ER stress, a condition closely associated with inflammation and lipid metabolism dysregulation (Han and Kaufman, 2016). Similarly, an extracellular pool of PDIA1 is well-known to support early thrombus formation (Flaumenhaft et al., 2015) and has been associated with vessel patency (Bekendam et al., 2018) and cytoskeleton assembly regulation in vascular smooth cells (Tanaka et al., 2018). Furthermore, extracellular PDIA1 sustains an anti-constrictive remodeling effect during vascular repair (Tanaka et al., 2016). Interestingly, although such an effect can support improved flow distribution in vascular diseases, it can also be a marker of enhanced inflammation and a complication of natural atheromas (Pasterkamp et al., 2004). These contrasting roles of PDIA1 in atherosclerosis may, in part, be related to its cell-type-specific effects in vascular cells under healthy or pro-atherogenic conditions (Tanaka et al., 2016; Ping et al., 2017; Kim et al., 2018). Recently, it was demonstrated that PDIA1 loss-of-function in endothelial cells (ECs) results in increased oxidant generation, promoting cell senescence and impairing endothelium-dependent relaxation (Kim et al., 2018). At the same time, the knockdown of PDIA1 in SMCs prevents agonist-induced oxidant generation and cell migration (Pescatore et al., 2012), and PDIA1 deletion in platelets prevents thrombus formation.

Therefore, understanding specific mechanisms underlying the expression of PDIA1 in various vascular cells and how they interplay with processes involved in atherosclerosis evolution could provide relevant advances. Previously, we reported that the co-induction of PDIA1 and RhoGDIα, which form a highly conserved microsyntenic gene cluster, is highly correlated in the intimal (mainly endothelial) layer under pro-atherogenic flow conditions. At the same time, a similarly robust correlation was not observed in the other vessel layers (Moretti et al., 2017). This indicates that factors regulating PDIA1 and RhoGDIα levels, including potential microRNAs (miRs), could exert cell-specific effects relevant to vascular physiology. MiR-based regulation plays emerging roles in atherosclerosis biology, as indicated by effects mediated by cell-specific miRs and paracrine regulation (Kumar et al., 2014). There are several examples of miRs exerting anti, pro, and dual effects related to mechanisms of atherosclerosis, such as miR-145 (Hergenreider et al., 2012), miR-712 (Son et al., 2013), and miR-155 (Donners et al., 2012; Du et al., 2014), respectively. miR-145 was shown to target VSMC differentiation through its release via endothelium-derived vesicles (Hergenreider et al., 2012); miR-712 modulates endothelial layer permeability by targeting the inhibitor of metalloprotease TIMP-3 (Son et al., 2013); and miR-155 demonstrates either attenuation or upregulation of inflammation and atherosclerosis, following its manipulation in myeloid cells. In addition, miRs play specific roles in distinct phases of atherosclerosis. A major pro-atherogenic condition is a disturbed pattern of flow involving low and oscillatory shear stress (Kumar et al., 2014). Addressing PDIA1 expression regulation in this setting may provide clues to the roles of PDIA1 in atherogenesis modulation and help understand how redox processes modulate vascular diseases. Here, we investigated whether miR-dependent regulation of PDIA1 plays relevant roles in mechanisms associated with atherosclerosis development and whether such effects are vascular cell type-specific.

## Materials and methods

### Animal model of partial carotid ligation

Animal studies were performed with male C57Bl/6 mice according to the approved IACUC protocol by Emory University and by the Scientific Research and Ethics Committees of the Heart Institute and School of Medicine, University of São Paulo, Brazil, SDC number 3334/08/085 and CEUA (Animal Experiments Committee, protocol 1086/09). Mice were housed in the Division of Animal Resources at Emory University or the University of São Paulo School of Medicine, following a 12 h/12 h light/dark cycle with water and food *ad libitum.* In brief, anesthesia was induced initially with 5% isoflurane in oxygen and maintained at 1.5% throughout the procedure. Three of the four caudal branches of the left common carotid artery (LCA)—left external carotid, internal carotid, and occipital artery—were ligated with a 6–0 silk suture, while the superior thyroid artery was left intact (Nam et al., 2009). After 24 h, the blood flow was confirmed by ultrasound, and all analyzed LCAs showed low and oscillatory flow compared to the contralateral control right carotid artery (RCA), as reported (Nam et al., 2009). Mice were euthanized using CO_2_ asphyxia.

### Intimal RNA isolation from carotids

Total RNA from intima was separately obtained from LCA and RCA at 24, 48, or 72 h post-ligation, as described previously (Nam et al., 2009). In brief, LCA and RCA were quickly flushed with 150 μL of QIAzol lysis reagent (QIAGEN) using a 29G insulin syringe into a microfuge tube. The eluate was then used for total intimal RNA isolation using the miRNeasy Mini Kit (QIAGEN). The remaining tissue representing the medial and adventitial layers (M + A) was also used to analyze mRNA and microRNA expression (Nam et al., 2009). The quality and specificity of RNA isolation were confirmed by increased levels of the endothelial marker CD31 (PECAM-1) and lower levels of vascular smooth muscle cells (VSMCs; SM22) and leukocyte markers (CD45) at the intimal RNA elution (Supplementary Data).

### Prediction of miR(s) targeting PDIA1

Bioinformatic analyses with TargetScan (targetscan.org) were applied to assess potential miR(s) matching the PDIA1 gene sequence. First, P4HB (gene name of PDIA1, ENST00000331483) was entered as the input, and the broadly conserved miR among vertebrates was selected. MiRs and their predicted pairing with the P4HB sequence are presented in the “Results” section.

### Dual luciferase activity assay

Firefly luciferase activity was measured at room temperature with the Luc-Pair miR Luciferase Assay Kit (GeneCopoeia) using a single-tube luminometer (Model TD 20/20 Turner Designs, Sunnyvale, CA). Dual luciferase reporter constructs containing the 3′- untranslated region (UTR) of the *PDIA1* gene with miR-204-binding sites 5′-(AAAGGGA)-3′ (GeneCopoeia) or 3′-UTR of *PDIA1* with mutated miR-204-binding site 5′-(TCGATTC)-3′ (custom cloned at Emory Molecular Biology Core Facility) were transfected into human umbilical vein endothelial cells (HUVECs) using a Nucleofector Kit (Lonza). HUVECs were transfected first with wild-type or mutated target gene 3′-UTR using the HUVEC Nucleofector Kit (Lonza) and were allowed to recover for 24 h. The second transient transfection was performed using increasing concentrations of miR-mimic-204 or respective mismatch controls using Oligofectamine 3000 (Invitrogen). Firefly and Renilla luciferase activities were measured using a Luc-Pair miR Luciferase Assay (GeneCopoeia) as per the manufacturer’s recommendations.

### Quantitative real-time PCR

The total RNA of each sample was reverse transcribed into cDNA using SuperScript III and random primers (Invitrogen), as described (Nam et al., 2009). Alternatively, microRNA was transcribed into cDNA using miRNA SuperScript (Invitrogen). In brief, quantitative real-time PCR (qPCR) was performed on selected genes using Brilliant II SYBR Green qPCR Master Mix (Stratagene) with custom-designed primers on a Real-Time PCR System (ABI StepOne Plus). qPCR results were normalized based on 18S RNA expressions for mRNA and on U6 for microRNA. Fold changes between LCA and RCA were determined using the 2^−ΔΔCt^ method. The right carotid arteries were used as the internal controls, using either the respective RCA or the mean of the RCA analyzed in the same run.

### Immunofluorescence

For face staining, the arterial segment was carefully opened on the longitudinal axis, and the staining protocol performed similarly to the cryosectioned specimens. In brief, the slices were fixed in 4% paraformaldehyde for 20 min, permeabilized with 0.1% Triton-X for 10 min, blocked with 1% goat serum for 30 min, and then incubated overnight with specific primary antibodies against PDIA1 (C-terminal Enzo or RL90 Thermo). After three washes with phosphate buffered saline (PBS), the secondary fluorescent antibody was incubated with the nuclei marker DAPI for 1 h, and slices were mounted with the mountain medium (DAKO). For *en face* confocal measurement, 1 µm Z-stacks were performed, starting from intima to adventitia. Negative controls were performed by pre-incubating segments with the peptide designed against the last C-terminal 11 amino acids of the rat PDIA1 sequence in 5-fold excess vs. primary antibody. The quantification of PDIA1 expression in cross-sectional slices was performed, as described previously (Burgess et al., 2010).

### Transient transfection with miR-204 mimics *in vitro* and *in vivo*

HUVECs (BD Biosciences), used from passages 5 to 9, were cultured in M199 media (CellGro) with the following supplements: 20% fetal bovine serum (FBS, HyClone), 1% bovine brain extract, 10 mM L-glutamine, and 0.75 U/ml heparin sulfate, as described previously (Ni et al., 2011). HUVECs were transfected with control or miR-204 mimic 20 nM using oligofectamine, following the manufacturer’s instructions. After 24 h of transfection, the miRs and gene targets were evaluated. Primary vascular smooth muscle cells from the rabbit aorta were cultivated through the partial digestion protocol, as previously described (Tanaka et al., 2016), maintained in Dulbecco′s modified Eagle′s medium (DMEM) with low glucose supplemented with 10% FBS, antibiotics (penicillin/streptomycin), and used from passages 10 to 15. miR-204 mimic was transfected under serum- and antibiotic-free conditions and analyzed after 24 h in DMEM without serum. Rabbit aortic smooth muscle (RASM) cells permanently containing the PDIA1 overexpressing system were conditioned with doxycycline (dox) (Fernandes et al., 2021), pretreated or not with 1.5 μg/mL dox during 24 h, and then transfected with miR mimic control or miR-204, as described above. RASM cells were cultured with F-12 media supplemented with 10% FBS and antibiotics and were used until they reached passage 10. For *in vivo* transfection, miR-204 mimic was prepared in pluronic gel 30% by adding 5 µg of control or miR-204 mimic and transfection reagent (jetPEI). Sixty microliters of liquid cold gel were perivascularly applied, and analyses were performed after 48 h.

### Shear system *in vitro*

HUVECs seeded in gelatin 1% were used until they reached passage 9. Cells when 100% confluent were subjected to low-oscillatory (OS, 5 dynes/cm^2^ at 1 Hz each direction) or laminar shear stress (LS, 15 dynes/cm^2^) (14) during different times (3, 6, 12, 24, or 48 h).

### Western analysis

Arterial segments were lysed in RIPA buffer containing protease inhibitors. Twenty micrograms of proteins were analyzed under reducing and denaturing conditions. Primary antibodies were anti-PDIA1 (mouse, clone RL90, Thermo Fisher Scientific, 1:1000) and beta-actin (mouse, Sigma, 1:5000). Secondary fluorescent antibodies from LI-COR were detected using the Odyssey scanner with the 800 nm-channel for the mouse and 700-nm channel for the rabbit antibody and molecular mark detection.

### Collagen measurements

Arteries were fixed in PFA 4% for 20 min at room temperature (RT), washed with PBS, included in an optimal cutting temperature compound, and sectioned (10 µm) in a cryostat. Sections were maintained at RT for 20 min before proceeding with picrosirius red staining. Images were captured in 40× objective without and using a circular polarized filter to detect red–orange fibers, representing high birefringent (HB) fibers, which depict more mature and rigid collagen, usually attributed to type I, and low birefringent (LB) fibers appearing in green, depicting less rigid collagen, usually attributed to type III. In brief, using the RGB filters through ImageJ, the relative area of red–orange or green collagen was calculated specifically at the media and adventitia. Light exposure was longer for collagen at media vs. adventitial detection (Tanaka et al., 2016). For each sample, four distinct areas were acquired and quantified in a blinded way.

## Statistics

The results are presented as the means ± standard error, and comparisons were performed using a paired *t*-test or one-way ANOVA, followed by a Newman–Keuls *post hoc* test using GraphPad Prism 6.0. Statistical significance was determined at *p* < 0.05.

## Results

### Disturbed flow *in vivo* promotes PDIA1 expression

**FIGURE 1:**
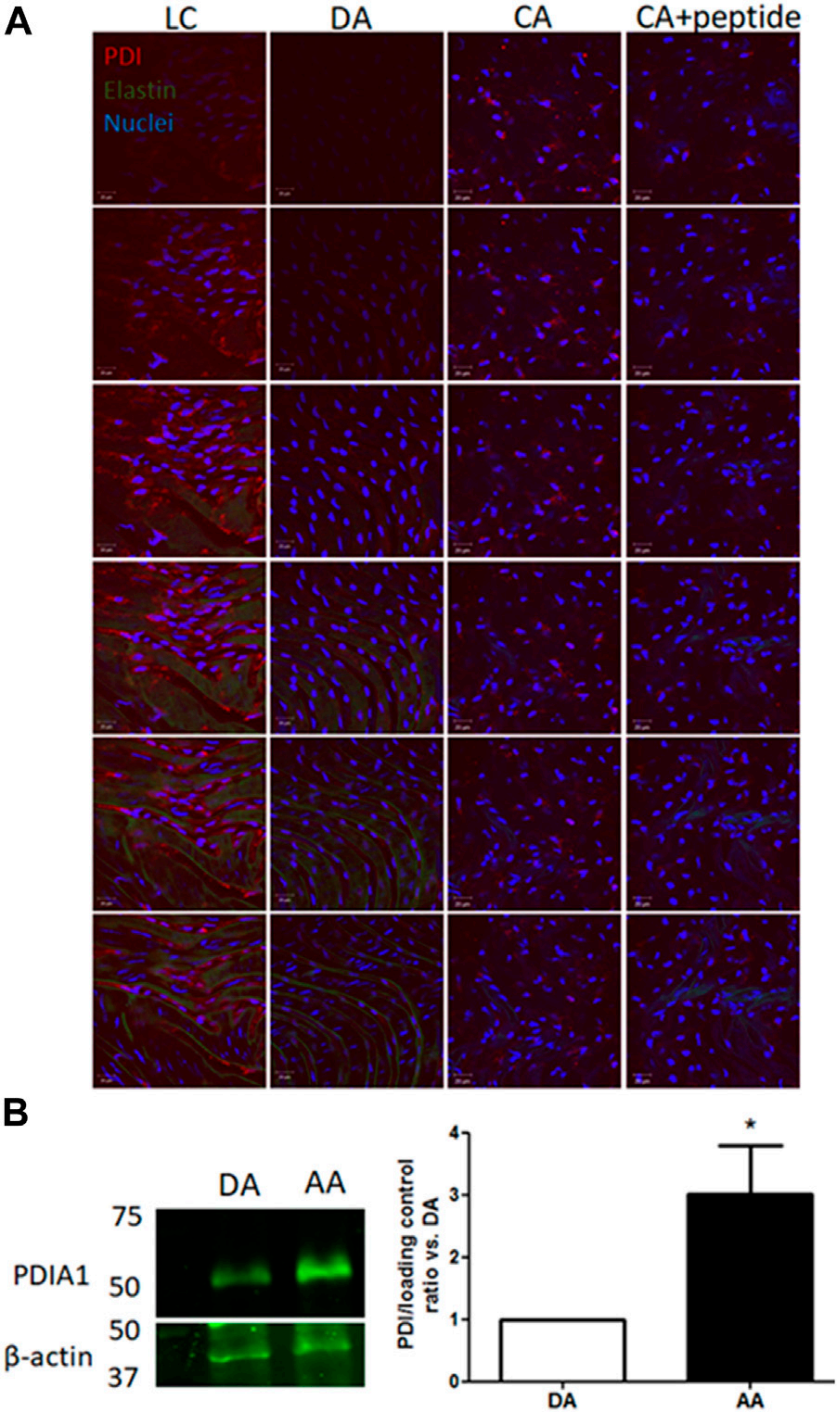
PDIA1 expression is increased in arterial regions exposed to disturbed flow. **(A)** *En face* immunofluorescence staining for PDIA1 in C57/Bl6 mouse arteries (from left to right): lesser curvature from aortic arch (LC), descending thoracic aorta (DA), control common carotid artery (CA), and control common carotid artery with blocking peptide against rat PDIA1 C-terminal 1:40 (CA + peptide). Images depict top-to-down 1 µm z-scan slices. Red, green, and blue staining depict PDIA1, elastin autofluorescence, and nuclei, respectively. Scale bar, 20 μm; *n* = 3, except for peptide control (*n* = 2). **(B)** Representative Western blot detecting PDIA1 in the descending aorta (DA) or aortic arch (AA) in the wild type mouse. Graph depicts the ratio of PDIA1 normalized vs. descending thoracic. β-actin or RhoGDIα was used as the loading control. *p < 0.05 vs. descending thoracic aorta, n = 3. The full membrane is presented in Supplementary Figure S1.

First, to investigate whether PDIA1 is differently expressed in vascular regions experiencing disturbed blood flow, expression levels of PDIA1 were determined by *en face* immunofluorescence comparing arterial locations well-characterized by stable, protective flow in the descending thoracic aorta (DA) and common carotid artery (CA) and disturbed atherogenic blood flow in the lesser curvature (LC) of the aortic arch. As shown in Figure 1A, the endothelial cells exposed to disturbed flow in the LC expressed significantly higher levels of PDIA1 than the stable flow regions (DA and CA). The specificity of the PDIA1 staining was demonstrated by the significant loss of antibody staining by the antigenic peptide competition used to develop the antibody (+peptide). Western blot results confirmed the higher amount of PDIA1 in the aortic arch (AA) containing the LC region than the DA (Figure 1B), indicating that naturally protected areas with stable blood flow patterns express lower levels of PDIA1 than the disturbed flow regions prone to atherogenesis.

**FIGURE 2:**
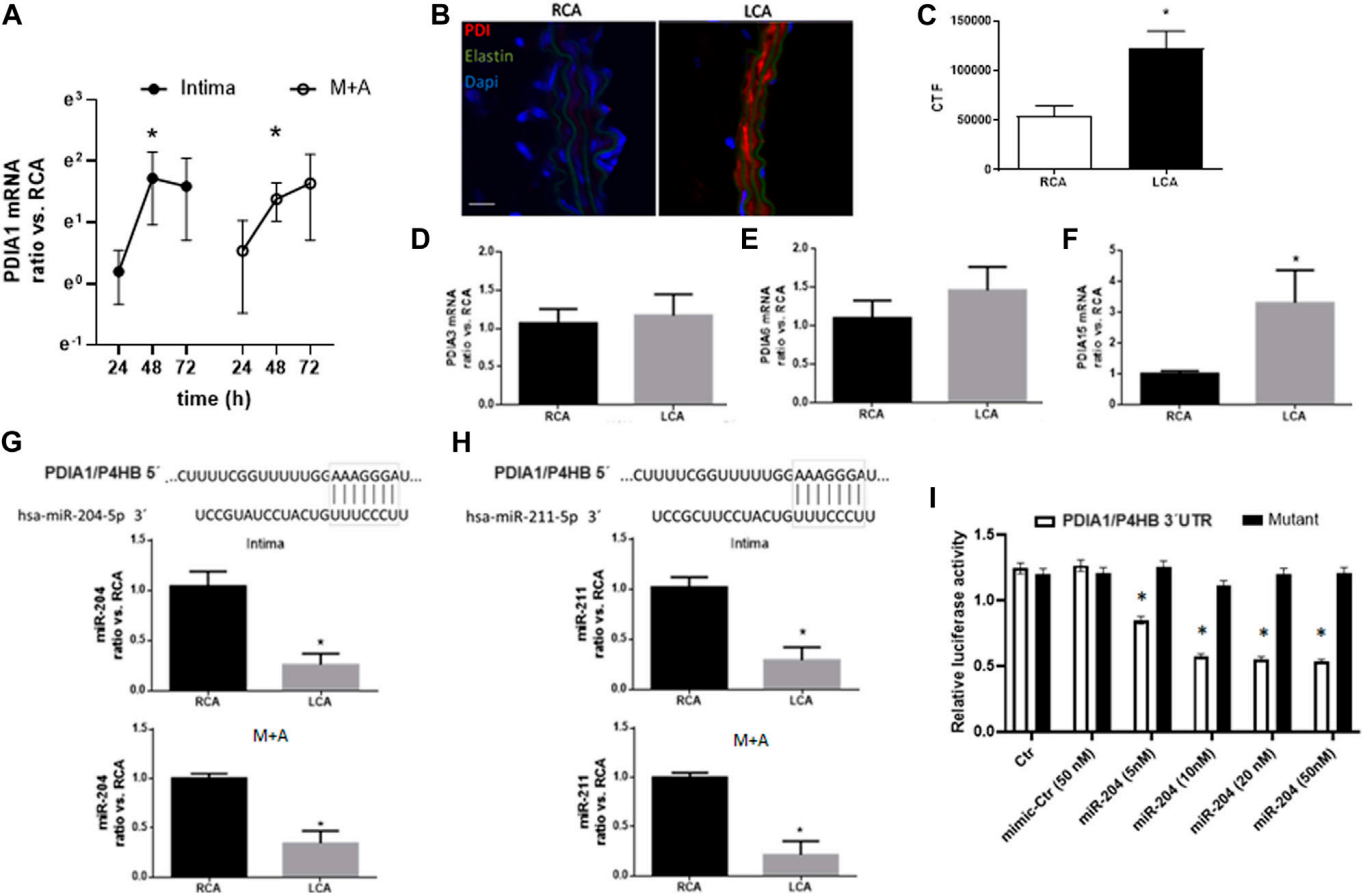
Disturbed flow increases PDIA1 expression while decreasing expression of miR-204 and miR-211 in the carotid artery. **(A)** Time course of PDIA1 mRNA expression at 24, 48, and 72 h, following PCL of the left common carotid artery (LCA) in intima and media + adventitia (M + A) layers. Data depicting the ratio of LCA vs. the contralateral right common carotid artery (RCA) control were log-normalized (base e) to ensure normal distribution. *p < 0.05 vs. RCA, *n* = 3–8. Validation for purity of each vessel layer is presented in Supplementary Figure S2. **(B)** Immunofluorescence of PDIA1 48 h after PCL. **(C)** Graph depicts immunofluorescence quantification expressed as corrected total fluorescence (CTF). **(D–F)** mRNA expression of additional PDI family members analyzed in the intima of RCA control or 48 h after PCL in the LCA: PDIA3, PDIA6, and PDIA15 (or endoPDI), respectively. Please note that PDI family members’ graphs are in different scales. *p < 0.05 vs. RCA, *n* = 6–8. “**B**”: Red, green, and blue staining depict PDIA1, elastin autofluorescence, and nuclei, respectively. Scale bar, 20 μm; *n* = 3. **(G, H)**: Top panels represent the conserved 3′UTR sequence from the PDIA1 gene (*P4HB*) paired with miR-204-5p (left) and miR-211-5p (right). Graphs represent miR-204 expression (left) and miR-211 (right) in intima (top) and M + A (bottom) from the RCA and LCA at 48 h post-PCL. *p < 0.05 vs. RCA, *n* = 5–6. **(I)** Dual luciferase assay for the 3′UTR sequence of *P4HB* (PDIA1 gene name) or a mutant sequence in HUVEC treated with miR mimic control (Ctr) or miR-204 mimic in different concentrations (5, 10, 20, and 50 nM). Graph depicts the relative activity of firefly corrected for Renilla luciferase. *p < 0.05 vs. both Ctr and mimic-Ctr, *n* = 3.

Next, we investigated acute molecular signaling mechanisms triggered by the disturbed flow. Previously, we reported that endoplasmic oxireductin 1 beta (ERO1L), a functional partner of PDIA1, which acts as a PDIA1 oxidase, was upregulated by disturbed flow 48 h after PCL (Ni et al., 2010). At the same time point (48 h) of such ERO1L induction (Ni et al., 2010), PDIA1 mRNA expression was increased in both the intimal (mainly endothelial) and medial plus adventitial layers (M + A) (Figure 2A) of the partially ligated left carotid artery vs. the control right carotid artery. Earlier (24 h) or later (72 h) measurements of *PDIA1* mRNA (Figure 2A) showed variable and non-significant differences compared to 48 h. The detailed description of the isolation of intimal and M + A mRNA is presented in Methods, and confirmation of the purity for each segment is shown by markers of specific layers, *PECAM* and *SM22*, for endothelium cells and vascular smooth muscle cells, respectively, and negligible immune cell infiltration is depicted by the expression of a macrophage marker (Supplementary Figure S2). In addition, PDIA1 protein expression at distinct vessel layers was increased 48 h after PCL (Figures 2B,C and zoom images in Supplementary Figure S3). Therefore, PDIA1 mRNA expression and protein are increased in the model of PCL, indicating the association of PDIA1 with an experimental model relevant to atherosclerosis development.

The PDI family protein contains over 20 family members in mammals, presenting variable conservation levels and functional similarities (Laurindo et al., 2012). To gain insight into whether PDIA1 induction was specific to PDIA1 during adaptation to the PCL model, other PDI family members were analyzed (Figures 2D–F). Of note, the mRNA expressions of PDIA3 and PDIA6 were not affected, but PDIA15 mRNA levels were increased. Thus, PDIA1 induction is not restricted to this family member, although it is not broadly extended to all family members.

### Disturbed flow *in vivo* downregulates miR-204/211 expression

Since microRNAs (miRs and miRNAs) are well-known to negatively regulate gene expression at the post-transcriptional level, we investigated whether miRNA-dependent mechanisms were playing a role during PDIA1 induction. From our bioinformatics screening approaches, we searched for a set of miRNAs that directly targeted PDIA1. *TargetScan* analysis for conserved miRs that potentially bind to the 3′UTR sequence of PDIA1 showed that miR-204 and miR-211 directly target PDIA1 (Figures 2G,H, top). Interestingly, both miRNAs (miR-204-5p and miR-211-5p; here, miR-204 and miR-211) are from the family of miR-204 and are derived from intron 6 of the transient receptor potential nonselective cation channel (TRPM) gene, with miR-204 from the *TRPM3* gene and miR-211 from TRPM1 (Chitnis et al., 2012). In order to validate the interaction between miR-204 and 3′UTR of PDIA1, HUVECs were transfected with the dual luminescent reporter containing the targeting or mutant sequence of 3′UTR of PDIA1. Indeed, all concentrations tested effectively decreased the luminescence of the reporter compared with the top concentration of the mimic control (Figure 2I). Importantly, both miRs were downregulated 48 h after PCL in the whole vessel (Figures 2G,H, bottom). Therefore, we speculate that the downregulation of these miRNAs by disturbed blood flow could be a potential mechanism for the induction of PDIA1 during vascular pathophysiological conditions resulting from disturbed blood flow (such as atherosclerosis and peripheral arterial disease).

### miR-204 targets PDIA1

**FIGURE 3:**
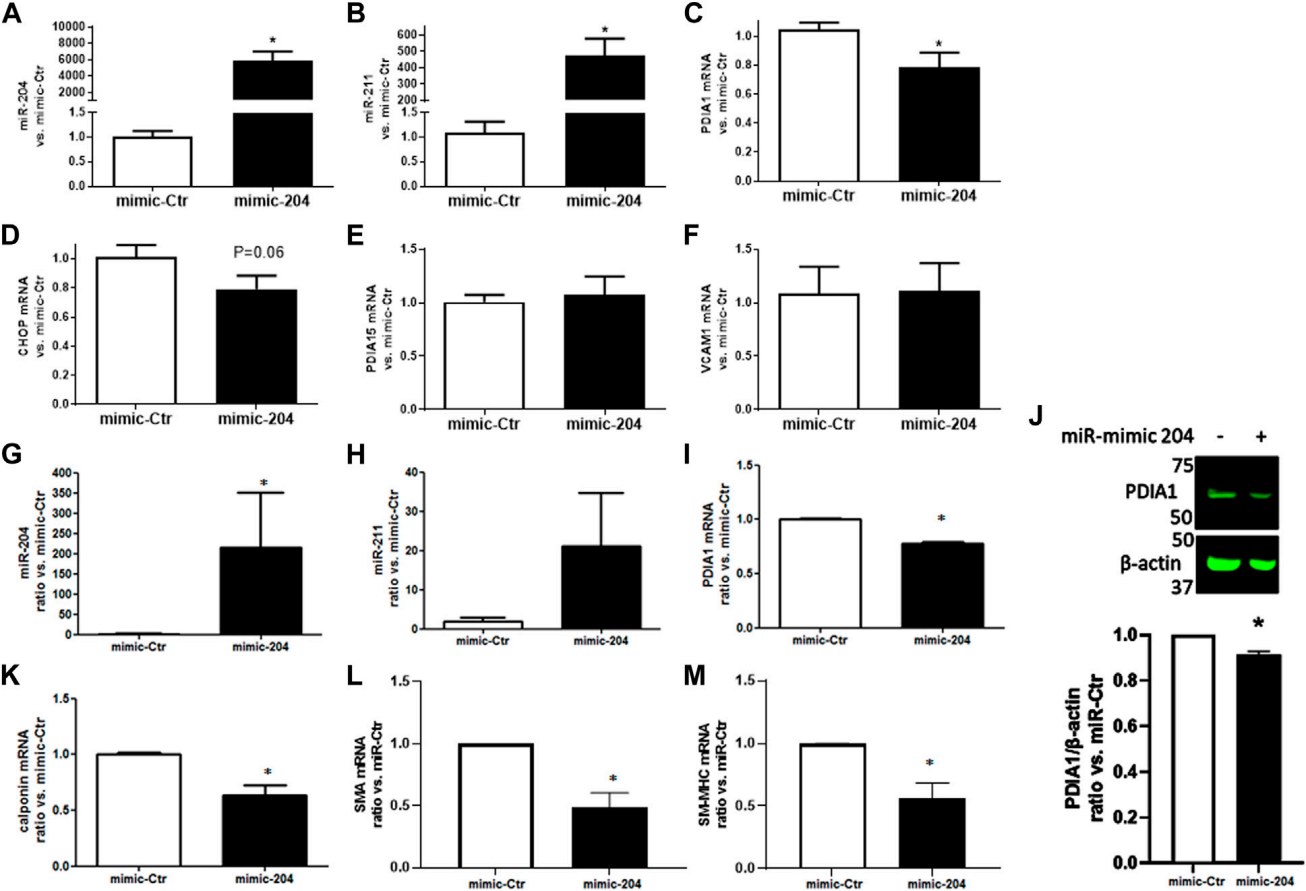
miR-204 mimic attenuates PDIA1 expression in endothelial cells and VSMC and reduces markers of VSMC differentiation. HUVECs **(A–F)** or rabbit aortic VSMC **(G–M)** were transfected with mimic control (mimic-Ctr) or miR-204 mimic (mimic-204) for 24 h, and expression of miR-204/211 and the target genes were analyzed by qPCR: miR-204 **(A and G)**, miR-211 **(B and H)**, *PDIA1* **(C and I)**, *CHOP* **(D)**, *PDIA15* **(E)**, *VCAM1* **(F)**, *calponin* **(J)**, *SMA* **(K),** and *SM-MHC* **(L)**. *p < 0.05 vs. mimic-Ctr, *n* = 3–4. PDIA1 protein expression in VSMC exposed to miR-mimic Ctr or miR-204 **(J)**. β-actin was used as the loading control. Graph depicts ratio vs. miR-Ctr of the relative PDIA1 expression. The full membrane is presented in Supplementary Figure S4.

To further investigate whether miR-204/211 plays a role in PDIA1 regulation, the gain-of-function experiments were conducted using miR mimics. HUVECs were transiently transfected with miR-204 mimics. Twenty-four hours after transfection, the expressions of both miR-204 and miR-211 were strongly upregulated, showing transfection efficiency (Figures 3A,B, respectively). Although only the miR-204 sequence was transfected, the concomitant increase in miR-211 could reflect either a cross-detection due to its high homology or positive feedback triggered by forced miR-204 induction. Importantly, PDIA1 (Figure 3C) and other targets of these miRs, such as CHOP (Figure 3D), were downregulated under basal conditions by miR-204 transfection, while PDIA15 and VCAM1 presented a negligible effect (Figures 3E,F). Furthermore, in primary rabbit aorta smooth muscle cells, miR-204 mimic transfection induced similar effects in miR-204/211 (Figures 3G,H), PDIA1 mRNA (Figure 3I), and protein expression (Figure 3J) and affected the markers of VSMC phenotype differentiation *calponin* (Figure 3K), smooth muscle actin (*SMA*, Figure 3L), and smooth muscle myosin heavy chain (*SM-MHC*, Figure 3M), which are not predicted to be directly targeted by miR-204/211.

### miR-204 gain-of-function prevents PDIA1 induction by disturbed flow and impacts the VSMC phenotype switch

**FIGURE 4:**
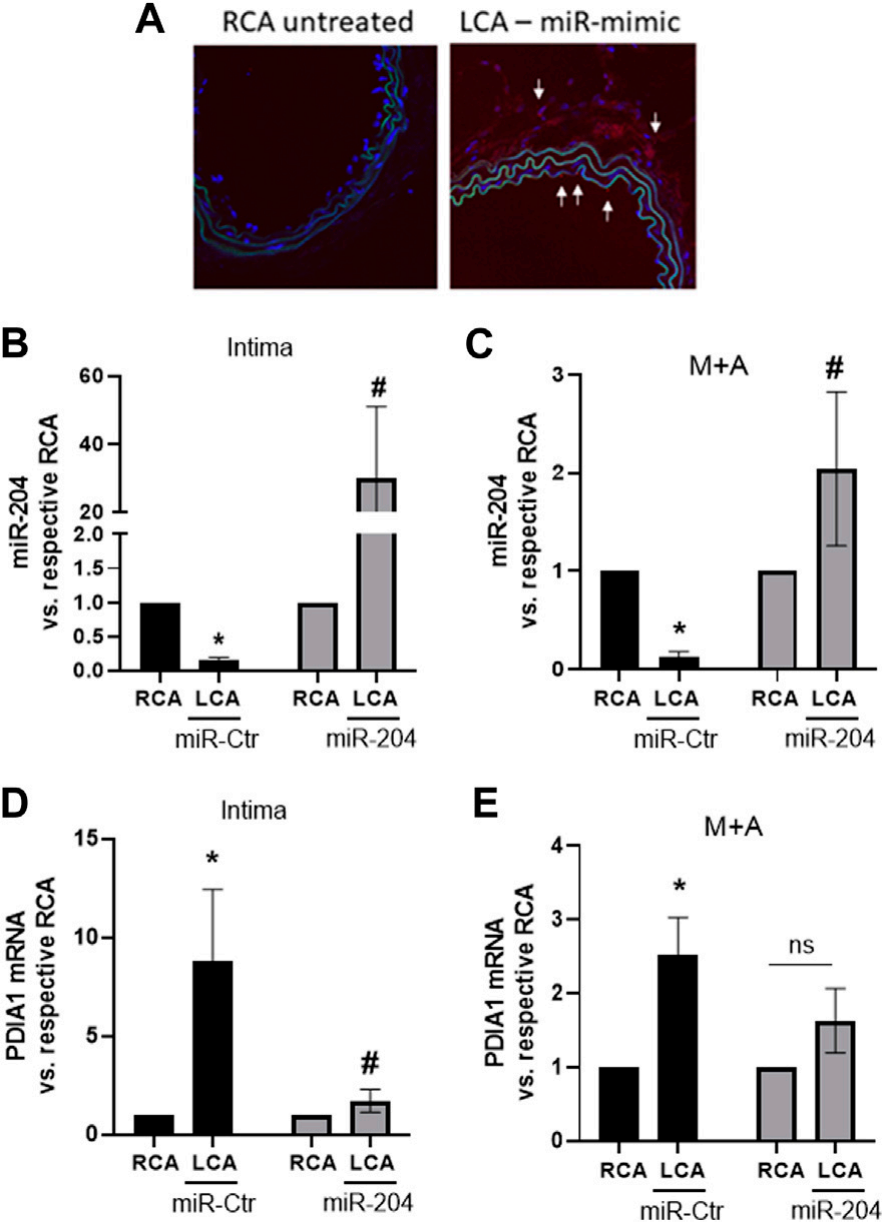
miR-204 mimic prevents PDIA1 upregulation by disturbed flow in the mouse LCA. **(A)**: miR-mimic delivery to the LCA was shown by fluorescent-labeled control miR-mimic (5 µg) delivered using pluronic gel (30%) *via* perivascular application for 48 h. Untreated RCA was used as a control. **(B–E)**: LCA was treated immediately after PCL surgery with mimic-Ctr or miR-204 mimic and RCA was maintained as control. Analyses were performed 48 h after PCL and miR-mimic treatments. **(B, C)**: miR-204 expression in the intima and M + A, respectively. **(D, E)**: *PDIA1* mRNA expression in the intima and M + A, respectively. All panels: miR or mRNA expressions are normalized by their respective RCA. *p < 0.05 vs. respective RCA and #*p* < 0.05 vs. LCA mimic-Ctr; *n* = 3–5.

To address the viability of efficiently transfecting miR mimic *in vivo*, a control fluorescent miR mimic (5 µg) was perivascularly applied to the common left carotid artery of control non-ligated mice. Indeed, 48 h after treatment, there was marked fluorescent staining in the adventitial layer as well as in additional locations close to the intima and media of treated arteries (Figure 4A, white arrows). Furthermore, following the application of miR-204 mimic in the PCL model, the downregulation of miR-204 in the LCA was prevented, with effects extending up to the intimal layer, in which there was a further increase in this miR (Figures 4B,C). Importantly, miR-204 mimic prevented the induction of PDIA1 by disturbed flow in both the intima (Figure 4D) and M + A (Figure 4E).

To test whether miR-204/PDIA1 plays a direct role in vascular inflammation, we determined the levels of pro- and anti-inflammatory molecules in the vessel wall, such as vascular cell adhesion molecule (VCAM-1) and Kruppel-like factor-2 (KLF-2) (Nam et al., 2009), respectively, upon treatment with miR-204 mimics. However, they were not affected by the miR-204 mimic (Supplementary Figure S5), suggesting that the miR-204/PDIA1 axis does not regulate vascular inflammation. In addition, CHOP, a key mediator of ER stress-dependent apoptosis, was downregulated, while PDIA15 expression was upregulated by disturbed flow. However, neither CHOP nor PDIA15 showed significant changes by miR-mimic 204 treatment vs. partially ligated arteries treated with miR-mimic control (Ctr). Thus, the reduction of PDIA1 by miR-204 overexpression does not markedly affect the endothelial response to the pro-atherogenic flow.

**FIGURE 5:**
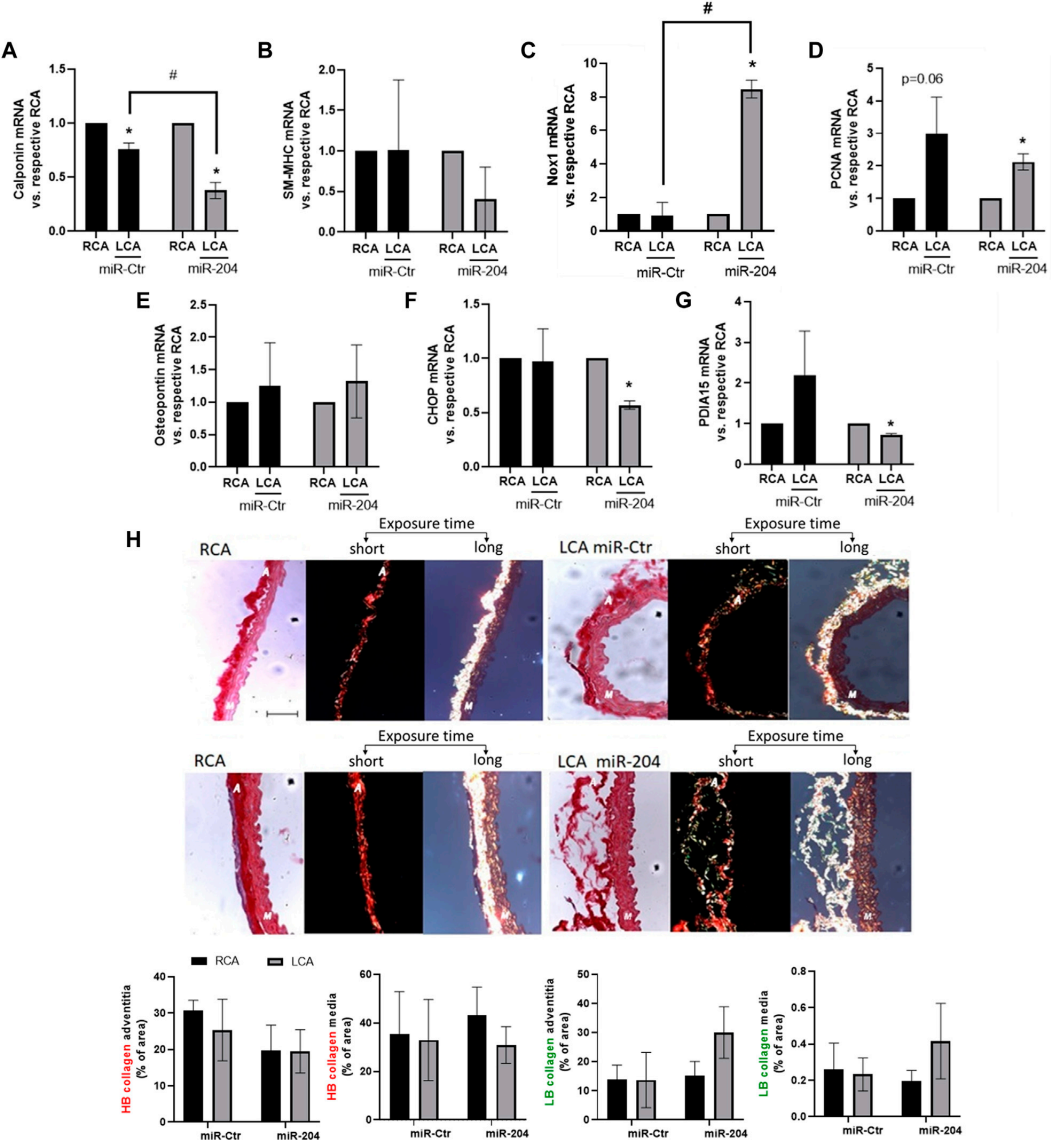
miR-204 mimic accentuates VSMC dedifferentiation by disturbed flow. Immediately following PCL, LCAs were treated with mimic-Ctr or miR-204 mimic (5 µg each) as shown in Figure 4 and RCA was maintained as the control. Analyses were performed 48 h after PCL and miR-mimic treatment for mRNA levels or collagen–extracellular matrix (ECM) architecture. The expression of *calponin* **(A)**, *SM-MHC* **(B)**, *Nox1* **(C)**, *PCNA* **(D)**, *osteopontin* **(E)**, *CHOP* **(F)**, and *PDIA15* **(G)** in M + A samples in the RCA and LCA was determined by qPCR. *n* = 3–5. mRNA expression is normalized to the RCA. *p < 0.05 vs. respective RCA; #*p* < 0.05 vs. LCA mimic-Ctr; *n* = 3–5. **(H)** Collagen–ECM was measured by light microscopy (of picrosirius staining) without (left) or with the circular polarizing filter with 40× magnification. Middle panels depict shorter exposure time during collagen measurement to visualize adventitial collagen. Right panels depict longer exposure time to measure collagen at the media layer. Scale bar = 50 µm. Adventitial and medial layers are represented by “A” and “M,” respectively, inserted in the images. Graphs at the bottom depict the relative content of high birefringent (HB), representing orange/red fibers or low birefringent (LB) collagen, representing yellow/green fibers, vs. area at adventitia or media layers measured using ImageJ.

Arterial regions exposed to disturbed flow are associated with the loss of the quiescent contractile phenotype of VSMCs (Long et al., 2011). Importantly, the VSMC differentiation marker calponin was downregulated by miR-204 treatment (Figure 5A), while SM-MHC (Figure 5B) was not significantly altered, neither by disturbed flow nor by miR-mimic 204. Interestingly, such a minor effect in the VSMC phenotype switch, as detected by decreased calponin, could involve Nox1 induction as its expression was accentuated by miR-204 mimic in the LCA (Figure 5C). Yet, PCNA induction by disturbed flow (Figure 5D) was not significantly affected by miR-204 mimic, and osteopontin (Figure 5E) did not change with disturbed flow or miR-204 mimic. Interestingly, CHOP and PDIA15 (Figures 5F,G) were downregulated by miR-204 mimic, indicating additional roles of miR-204 on endoplasmic reticulum (ER) homeostasis. As PDIA1 is involved with collagen processing, we evaluated the effect of miR-204 on collagen architecture in response to disturbed flow. Indeed, collagen fibers at the adventitia and media were not altered, neither by PCL nor miR-204 mimic treatment (Figure 5H). However, there was an increasing trend in the less rigid low birefringent (LB) collagen (suggesting type III collagen) in the adventitia and a decreasing trend in the more rigid high birefringent (HB) fibers (suggesting type I collagen) in the media in disturbed flow arteries treated with miR-204 mimic (Supplementary Figure S6). These results suggest that miR-204 does not broadly affect the responses to pro-atherogenic disturbed flow *in vivo* but causes minor effects on VSMC differentiation control and ER homeostasis.

### PDIA1 overexpression prevents miR-204 effects on VSMC phenotype regulation

**FIGURE 6:**
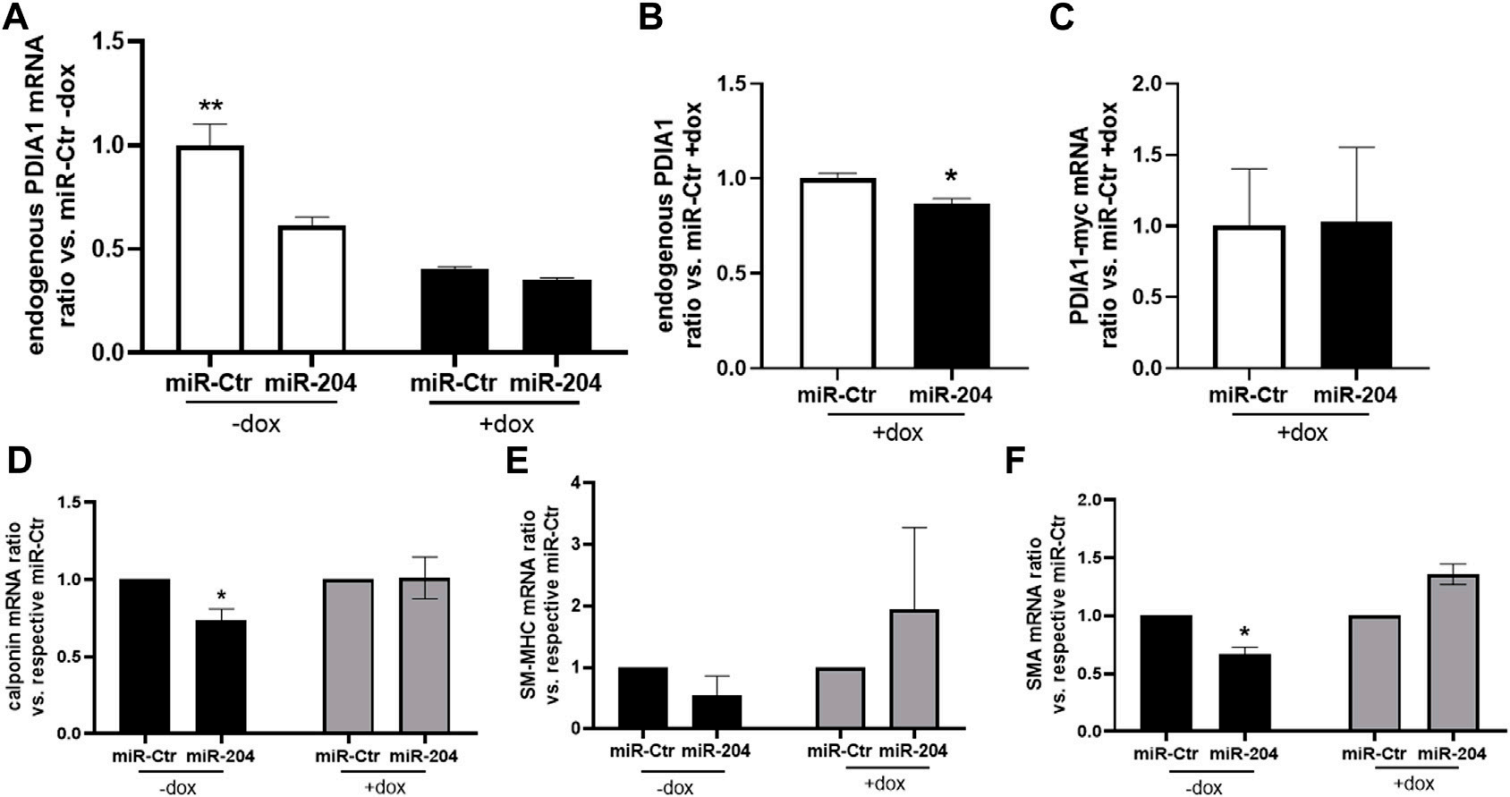
PDIA1 overexpression prevents loss of differentiation promoted by the miR-204 mimic. **(A–E)** Rabbit aortic VSMCs containing Tet-On sequence connected with rat PDIA1 tagged with myc sequence (PDIA1-myc) were pretreated (+) or not (−) with doxycycline (dox, 1.5 mg/mL) during 24 h, followed by miR-mimic transfection, as described in Figure 3. mRNA levels of endogenous PDIA1 normalized by miR-Ctr without **(A)** or with **(B)** doxycycline, exogenous *PDIA1-myc* **(C)**, *calponin* **(D)**, *SM-MHC* **(E)**, and *SMA* **(F)** were measured 24 h after transfection in the presence or absence of dox. N = 3–4. **p < 0.05 vs. other groups; *p < 0.05 vs. respective miR-Ctr.

The effect of PDIA1 counteracting VSMC dedifferentiation was reinforced in a cell model with conditional overexpression of myc-tagged PDIA1 (myc-PDIA1). Interestingly, either doxycycline (dox) incubation, which activates exogenous PDIA1 upregulation, or miR-204 mimic decreased endogenous PDIA1 mRNA levels (Figures 6A,B). Indeed, PDIA1 is reported to have a long half-life (Terada et al., 1995); thus, the downregulation of endogenous PDIA1 mRNA by forced expression of exogenous PDIA1-myc is likely to result from a crosstalk between distinct levels of control to maintain the homeostasis of PDIA1. Yet, myc-PDIA1 was detected only in dox-treated VSMCs (Figure 6C) and was not significantly affected by miR-204 mimic. Such a negligible effect is expected, as the sequence for PDIA1 induction contains only 11 nucleotides from 3′-UTR, thus making it unlikely to be targeted by miR-204. At the protein level, miR-mimic 204 attenuated the expression of PDIA1 (Supplementary Figure S7). Such a decrease was detected only without PDI overexpression (-dox), probably reflecting the incapacity to distinguish endogenous from exogenous PDIA1 by the antibody used for PDIA1 measurement (RL90). Importantly, myc-PDIA1 expression inhibited the downregulation of calponin and SMA (Figures 6D,F). In this case, miR-204 upregulation did not alter SM-MHC expression (Figure 6E), which could be due to the fact that such cells are not from primary culture (vs. Figure 3L). Overall, these findings are in line with recent studies from our group showing that PDIA1 is an upstream organizer of VSMC plasticity (Fernandes et al., 2021) and highlight a potential protective role of PDIA1 in arteries under disturbed flow conditions.

## Discussion

In the present study, we observed that PDIA1 is strongly expressed in areas with either naturally or induced disturbed flow and that the downregulation of miR-204/211 plays a role in PDIA1 expression. In addition, we detected that PDIA1 induction correlates with the attenuation of VSMC dedifferentiation during the early phase of vessel response to disturbed flow. Such a protective role is a novel finding, further underscoring the role of PDIA1 in the modulation of the VSMC phenotype, a fundamental process in the genesis of vascular disease. Given the central role of PDIA1 in ER homeostasis and its emerging roles in cell signaling (Laurindo et al., 2012), these findings may have implications for disclosing novel redox-related anti-atherosclerotic mechanisms.

The loss of VSMC differentiation and migration contributes to wall thickening through neointima or plaque development (Bennett et al., 2016). Important roles of microRNA-dependent regulation of the VSMC phenotype have been widely reported, ranging from embryonic development to vascular diseases (Albinsson et al., 2010; Tang et al., 2017). Indeed, the gain-of-function of microRNAs associated with VSMC differentiation prevents neointima formation, as is the case of miR-145 (Cheng et al., 2009) and miR-21 (Ji et al., 2007), while miR-221 (Liu et al., 2009) inhibition results in similar endpoints involving specific pathways regulating VSMC plasticity. Interestingly, miR-204 has also been reported to promote VSMC dedifferentiation by downregulating caveolin-1 (Torella et al., 2018). Here, we reported that miR-204/211 is involved in VSMC dedifferentiation, at least in part by targeting PDIA1 expression. The primary aim of this study was to address a mechanism affecting PDIA1 through microRNA dynamics; therefore, it is more feasible to investigate shortly after the induction of disturbed flow. Even under normal conditions, the medial layer depicts VSMC at distinct degrees in the continuum between synthetic(s) and full contractile phenotypes (van Kuijk et al., 2019). In addition, although the VSMC phenotypic switch is a common process in several cardiovascular diseases, such changes are model-dependent, and the profile of marker alterations does not follow strictly the same pattern of intensity and time course (Owens et al., 2004). Here, such complexity was reflected in the distinct responses to disturbed flow in the *in vivo* model and also in isolated cells, which, overall, depicted the loss of VSMC contractile markers by the miR-204 gain-of-function. Indeed, as the overexpression of PDIA1 prevented the effect of disturbed flow or miR-mimic 204, these results may suggest the role of PDIA1 in the resilience of VSMC phenotype maintenance.

There are several potential mechanisms by which PDIA1 could affect the balance of VSMC differentiation. In previous studies, we reported that PDIA1 closely regulates the biology of NADPH oxidases (Noxes), being required to sustain Nox1 agonist-induced activity and expression (Laurindo et al., 2014). In turn, Noxes have been distinctly involved with VSMC differentiation, with Nox4 supporting the maintenance of a contractile phenotype (Clempus et al., 2007), while Nox1 induction associates with the VSMC synthetic phenotype (Rodriguez et al., 2015) and neointimal thickening (Lee et al., 2009). Previously, we reported that silencing PDIA1 in a model of vascular repair to balloon injury accentuated the induction of VSMC dedifferentiation (Tanaka et al., 2016), which was recently demonstrated to be associated with Nox1 upregulation and Nox4 attenuation during repair to injury (Fernandes et al., 2021). Here, although Nox4 was not consistently detected (neither RCA nor LCA), Nox1 induction by disturbed flow was upregulated by miR-204 mimic, or else, reinforcing the potential of PDIA1 loss-of-function has led to Nox1 increases, thus accentuating the VSMC phenotype switch. Moreover, in transgenic mice constitutively overexpressing PDIA1, conductance arteries display an overdifferentiated the VSMC phenotype (Fernandes et al., 2021). The mechanisms for these effects are not yet clear, but the marked PDIA1 effects on cytoskeletal regulation and RhoGTPase activation (Tanaka et al., 2018), as well as on integrin activation (Tanaka et al., 2016; Araujo et al., 2017; Tanaka et al., 2018), are possible pathways. As the proliferative and osteogenic signaling markers PCNA and osteopontin, respectively, were not altered by miR-204 mimic in disturbed flow arteries, a reported null phenotype may have been induced, which is characterized by an undefined VSMC phenotype in the array of plasticity decisions, which was promoted by disrupting RhoGTPase signaling (Talwar et al., 2021). Accordingly, we reported that changes in PDIA1 expression closely correlate with those from RhoGDIα in carotid arteries under laminar and disturbed flow (Moretti et al., 2017). RhoGDIs are RhoGTPase chaperones mediating the directional and localized activation of RhoGTPases, which themselves closely regulate VSMC plasticity, with RhoA supporting differentiation (Lu et al., 2001) and Rac1 supporting the synthetic phenotype (Shi et al., 2014). Interestingly, we reported previously that the PDIA1-RhoGDIα correlation is more pronounced in the endothelial layer than in the M + A layer (Moretti et al., 2017). Here, such a positive correlation was attenuated by miR-204 treatment in the intimal layer and abrogated the significance in M + A (Supplementary Figure S8), likely because RhoGDIα is not targeted by miR-204. This, together with the effect of PDIA1 downregulation by miR-mimic transfection in endothelial cells and VSMCs, may help explain the disturbed capacity to control VSMC phenotypic modulation during response to disturbed flow.

The co-regulation between PDIA1 and RhoGDIα extends to additional genes from their families, represented by three pairs of PDI/RhoGDI arranged as microsyntenic clusters. Because we reported previously that such co-regulation is highly conserved up to the last common vertebrate ancestor, we investigated well-conserved miRs in vertebrates potentially regulating *P4HB* (gene name of PDIA1), which depicted microRNAs from the miR-204 family (204 and 211). However, a less restricting search depicts additional non-conserved miRs, including 205-5p, 10-5p, 383-5p.1, 140-3p.1, 212-5p, and 338-3p. However, none of them were found to be downregulated 48 h after PCL (Son et al., 2013). Beyond miR-204/211 presented here, miR-210 has been shown to play a role in PDIA1 regulation (Fasanaro et al., 2009; Lee et al., 2015). Specifically, in endothelial cells under hypoxia, the upregulation of miR-210 decreases PDIA1 levels (Fasanaro et al., 2009), while in glioblastoma multiforme, the low levels of miR-210 were positively associated with PDIA1 upregulation and a chemoresistance phenotype (Lee et al., 2015). Thus, we cannot exclude the potential interference of disturbed flow on the control of other microRNAs listed above and their impact on PDIA1 expression.

The primary and direct target of disturbed flow in the vascular system are mechanosensors in endothelial cells, which trigger mechanotransduction through several mechanisms reported to release paracrine mediators that affect VSMC phenotype control—including oxidants, proteins, and non-coding RNAs, as follows: first, nitric oxide (NO) bioavailability is decreased by pro-atherogenic blood flow, either by decreasing its production or increasing its inactivation by the generation of oxidants such as superoxide (Wang et al., 2019). Of note, the anti-proliferative action of NO on VSMCs has been described for a long time (Garg and Hassid, 1989). Importantly, Kanno et al. (2008) reported that NO prevents the induction of calcifying VSMCs by inhibiting TGF-beta signaling. Second, platelet-derived growth factor (PDGF), including PDGF-BB, has been reported to disrupt VSMC quiescence, stimulating migration, proliferation, and neointimal growth (Li et al., 2013). PDGF-BB is increased in endothelial cells after 48 h of partial carotid ligation (PCL; Mondy et al., 1997), being released by endothelial cells under flow stimulation (Palumbo et al., 2002) and stimulating the VSMC phenotype switch through mTOR signaling (Ha et al., 2015). Third, studies from distinct groups (Hergenreider et al., 2012; Zhou et al., 2013) have reported a role for microRNAs released from endothelial cells in controlling the VSMC phenotype through different pathways. Specifically, miR-143/145 is induced in endothelial cells by protective flow and released through extracellular vesicles, leading to VSMC contractile maintenance. Such protection is lost under disturbed flow conditions (Hergenreider et al., 2012). In contrast, miR-126-3p is increased in VSMCs due to its release from endothelial cells under static pro-atherogenic conditions vs. laminar protective flow, which induces VSMC proliferation (Zhou et al., 2013). Here, we describe for the first time the association of miR-204/211 downregulation with VSMC phenotype control.

Endothelial cells can regulate VSMC function through paracrine mechanisms, including microRNA secretion (Hergenreider et al., 2012). Here, our results do not highlight such a type of regulation since disturbed flow induced miR-204/211 downregulation in both the intimal and M + A layers, and PDIA1 expression was increased in both as well. However, the *in vivo* miR-204 transfection was more accentuated in endothelial cells vs. M + A, which also reflected in a more accentuated loss of PDIA1 expression. Such higher exogenous miR uptake was previously demonstrated to occur in endothelial cells subjected to disturbed flow. On the other hand, the paracrine role of endothelium-derived PDIA1 was recently shown to regulate platelet function (Bekendam et al., 2018); thus, we cannot, in principle, exclude that endothelial PDIA1 can control VSMC differentiation in a paracrine manner by miR-independent mechanisms. Interestingly, although the inflammatory markers were not differently affected by miR-mimic 204, the pattern of VCAM-1 distribution was changed by miR-204 upregulation (Supplementary Figure S9). Although VCAM-1 is not detected in RCAs, it was markedly increased in LCAs, and interestingly, it showed a distinct distribution comparing treatment with miR-mimic Ctr vs. 204. While the first was increased at the media, the later was more pronounced at the intima. To the best of our knowledge, such a distinct distribution of VCAM-1 has not been reported to be involved in the VSMC phenotype switch, which is the pathophysiologic endpoint induced by partial carotid ligation differentially modulated by the miR-204 mimic. To further investigate potential mechanisms involving endothelial-mediated paracrine regulation on VSMCs, we addressed endothelium-specific responses to flow using cell culture approaches based on laminar and disturbed flow. However, our results were contrary to those observed in our mouse studies. PDIA1 mRNA expression was downregulated by disturbed flow in a similar time course of KLF2 decreases and VCAM-1 increases (Supplementary Figure S10). This difference is not surprising, as a previous report showed approximately 31% divergence between the cell and. mouse-based models of disturbed flow using array analyses (Ni et al., 2010). Additionally, not only PDIA1 expression but also its function have been reported to diverge in *in vivo* and *in vitro* approaches (Tanaka et al., 2016; Araujo et al., 2017; Tanaka et al., 2018). Despite such model-dependent-responses, disturbed flow *in vitro* increased miR-204 expression, reinforcing its involvement in the regulation of PDIA1 levels. However, this gene/miR balance was not always observed, as PDIA1 mRNA upregulation by TNFα treatment in endothelial cells was disclosed to miR-204/211 alterations (Supplementary Figure S11).

miR-204 and miR-211 are derived from intronic regions of the cation channels TRPM3 and 1, respectively (Courboulin et al., 2011). Indeed, miR-204 exhibits highly tissue-specific expression, being enriched in several neural cells, including the kidney, testis, and retina (Liu et al., 2021). Courboulin et al. (2011) reported that TRPM-3 downregulation was associated with miR-204 decreases in pulmonary hypertension, although the role of miR-204 had been independent of TRPM-3 expression (Courboulin et al., 2011). The expressions of TRPM1 and 3 mRNA were not consistently detected in either the intimal or M + A layers (data not shown). In contrast, miR-204/211 is expressed at detectable levels, which could be attributed to the low levels of TRPM-1/3, as TRPM3 was reported to be targeted by miR-204 (Hall et al., 2014). miR-204 has been considered a tumor-suppressor microRNA, being negatively correlated with tumor malignancy (Yang et al., 2023). In the colorectal cancer cell line HCT116, a highly invasive and metastatic cell line, PDIA1 levels were shown to be increased compared with normal or less aggressive colorectal cancer cell lines (De Bessa et al., 2019). Accordingly, miR-204 is significantly downregulated in HCT116 cells (Wu et al., 2016). Yet, in several glioma cells, the decreased levels of miR-204 were associated with hypermethylation at the promoter CpG region of the TRPM3/miR-204 gene (Ying et al., 2013). At the same time point, we found here (48 h after PCL) that the expression of DNA methyltransferase-1 (DNMT1) was reported to be increased both at the intima and M + A layers (Dunn et al., 2014). As the inhibition of DNA methylation recovers miR-204 expression in cancer cells (Ying et al., 2013), this mechanism may be involved with miR-204 downregulation by disturbed flow. Alternatively, STAT3 activation has been shown to repress miR-204 expression in pulmonary artery smooth muscle cells (Courboulin et al., 2011) and cancer cells (Bao et al., 2013). In addition, STAT3 signaling is increased in VSMCs stimulated with pro-atherogenic treatment (Zhai et al., 2022); therefore, miR-204 downregulation could be associated with STAT3 overactivation. Overall, miR-204 expression is regulated by several levels of control (Liu et al., 2021), and some of them are associated with pathological conditions reported to present increased PDIA1 expression.

miR-204 mimic transfection also increased the detection of miR-211. Such an effect can result from their high similarity, resulting in an artefactual increase. The difficulties in clearly distinguishing between miR-204 and miR-211 were reported by other studies (Vitiello et al., 2017; Grieco et al., 2019). Beyond their detection, there is a large overlap of their targets, while the effects of inhibitors can also overlap, with one downregulating the other. All these issues make it hard to exclude potential cross-effects (Grieco et al., 2019). Indeed, both miR-204 and miR-211 depict similar anti-motility and anti-proliferative effects in melanoma cells (Vitiello et al., 2017), although their induction with antitumor compounds was differential. Thus, although the rule of similar miR families representing the same effects has limitations, we reported that both were downregulated by disturbed flow, while PDIA1 was upregulated. If there is any distinction in response to disturbed flow, it needs to be further explored. For instance, either miR-204 or miR-211 mimic could be used in gain-of-function experiments as their nucleotide sequences pairing with PDIA1 3′-UTR are the same. Indeed, the predicted targets provided by TargetScan do not distinguish between miR-204 and. miR-211. Such overlapping effects were reported previously (Lee et al., 2016), and rescuing only one miR was shown to be enough to overcome the phenotype induced by the double knockout of miR-204/211 (Huang et al., 2019). The distinct biological effects of miR-204 vs. miR-211 are related to conditions where only one of them is up or downregulated, such as in melanoma cells exposed to the chemotherapeutic drug vemurafenib (Vitiello et al., 2017). However, this was not the case for the present study, as both were downregulated by disturbed flow in the intima and M + A layers.

Importantly, miR-204 or miR-211 alterations have been reportedly involved in several pathophysiological mechanisms, including proliferation, apoptosis, and ER stress (Courboulin et al., 2011; Chitnis et al., 2012; Kassan et al., 2017). In the present study, genes involved in such pathways were not markedly and consistently sensitive to miR-204 mimic transfection, such as PCNA and CHOP. The latter, which is a master regulator in the apoptotic arm of ER stress, has been associated with plaque instability and was reportedly targeted by miR-204. Collectively, miR-204 and 211 presented a time-dependent expression profile in the model of balloon injury, which is not surprising due to the myriad of miR-204/211 targets that mostly follow a time-dependent modulation during injury repair (Ji et al., 2007). Previously, we reported that the peak of PDIA1 mRNA induction occurred 7 days after balloon injury (Tanaka et al., 2016), and at this time, miR-204 was shown to be downregulated (Ji et al., 2007). Although the effects of miR-204 mimic on PDIA1 mRNA expression in isolated endothelial and VSMC are discrete, such effects were addressed under baseline conditions. For instance, similar effects were detected at the protein level in distinct VSMCs. However, even presenting small effects, PDIA1 decreases promote measurable effects in VSMC biology, for example, impacting phenotype switch. In previous studies from our group, we observed that even minor decreases in PDIA1 expression by siRNA transfection promoted significant effects on target variables (unpublished data). In addition, a small decrease may be unlikely to affect the role of PDIA1 in protein folding, as it is found in mM levels at the endoplasmic reticulum; however, for distinct locations (assuming distinct functions), differences in nM order impact the cardiovascular pro-atherogenic phenotype in humans (Oliveira et al., 2019). Yet, in the model of disturbed flow *in vivo*, which is associated with miR-204 downregulation, the gain-of-function of miR-204 prevented PDIA1 increase by approximately 4- and 2-fold in the intima and M + A layers, respectively. Considering the roles of PDIA1 in thrombus formation (Flaumenhaft et al., 2015) and vessel patency (Kim et al., 2018) and its involvement in the maintenance of VSMC differentiation (Fernandes et al., 2021), further understanding miR-204/211 modulation in atherosclerosis may help elucidate mechanisms underlying responses to atherogenic stimuli and plaque vulnerability. Moreover, we cannot exclude a potential synergistic effect of the miR-204/211-PDIA1 axis with the modulation of another PDI family protein, such as PDIA15, which exerts effects similar to those of PDIA1 during response to TNFα and angiogenesis (Camargo et al., 2013), and both have been targeted in models of thrombotic diseases (Zhou et al., 2022). Here, we detected that PDIA15 is also upregulated in the model of disturbed flow. However, PDIA15 is not predicted to be downregulated by miR-204/211 and presented a negligible effect in isolated endothelial cells treated with miR-204 mimic, thus limiting the potential interference of PDIA15 on the effects directly caused by miR-204 upregulation.

**FIGURE 7:**
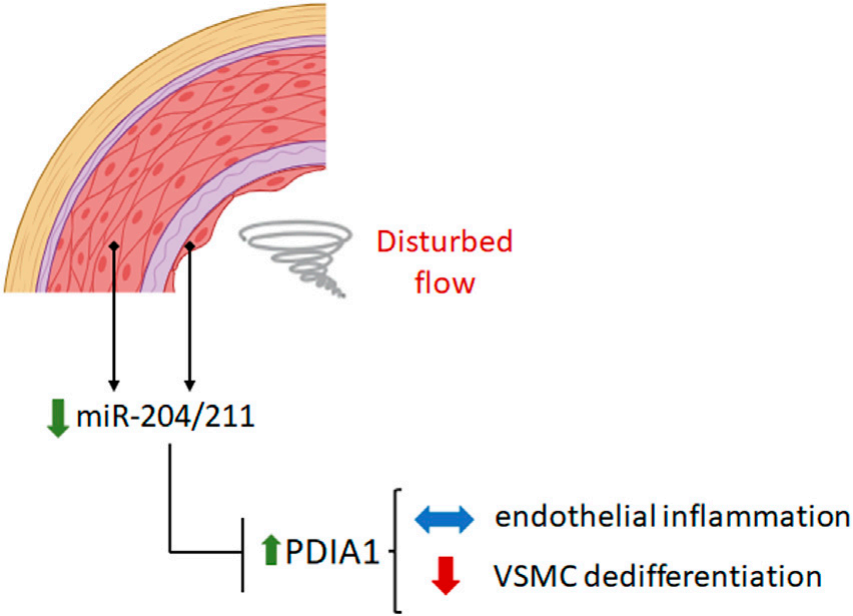
Disturbed flow downregulates miR-204/211 expression, which increases PDIA1 levels. Reducing the PDIA1 expression level by miR-204 mimic treatment does not affect endothelial inflammation but accentuates the VSMC phenotype switch under disturbed flow conditions. The figure was created by BioRender.com.

Overall, the present results (summarized in Figure 7) lend further support to our recently proposed PDIA1/redox model of vascular remodeling (Tanaka and Laurindo, 2017; Portas et al., 2022) while raising two new levels of regulation focusing on PDIA1 involvement: one upstream to PDIA1, mediated by miR-204/211, and the second downstream to PDIA1, affecting VSMC differentiation. These results corroborate possible roles of PDI as a central hub in the regulation of vascular redox homeostasis (Tanaka and Laurindo, 2017; Portas et al., 2022).

## Data Availability

The original contributions presented in the study are included in the article/Supplementary Material; further inquiries can be directed to the corresponding author.
