# Supplementary material for: Disturbed flow regulates protein disulfide isomerase A1 expression via microRNA-204
Source: Front Physiol. 2024 Apr 4;15:1327794. doi: 10.3389/fphys.2024.1327794 (PMC11024637; doi:10.3389/fphys.2024.1327794)
Supplement: Supplementary file 2 [file Presentation1.pdf]

Disturbed flow regulates protein disulfide isomerase A1 expression via microRNA-204

Leonardo Y. Tanaka[1]; Sandeep Kumar[2]; Lucas F. Gutierre[1]; Celso Magnun[1]; Daniela Kajihara[1]; Dong-Won Kang[2]; Francisco R.M. Laurindo[1]; Hanjoong Jo[2]

Legends of Supplementary Figures 1-11

Supplementary Fig. 1 Uncropped membrane presented at Figure 1B. PDIA1 was detected first and β-actin plus calponin were measured subsequently. Wild type (WT) and mice overexpressing PDIA1 (TgPDI) were measured in this experiment, but only WT[1] is shown. White box depicts selected lanes.

Supplementary Fig. 2 Control for purity of RNA isolation. Intimal (A) RNA extracted from carotid arteries are enriched in PECAM-1, while left over (B) is enriched in SM22 and both presented low levels of leukocyte marker (CD45). N=3.

Supplementary Fig. 3. Control carotid artery (RCA) or 48 h after partial carotid ligation (LCA) were stained for PDI (red) and nuclei (Dapi, blue), elastin autofluorescence is shown in green. Images are magnification from Figure 2B. External elastic lamina (EEL) and internal elastic lamina (IEL) are highlighted in white dotted line, luminal size is pointed as L and intimal PDIA1 labeling are pointed with yellow arrows, while medial PDIA1 in LCA is clearly detected between EEL and IEL. Scale bar 10 µm.

Supplementary Fig. 4. Uncropped membrane presented at Figure 3J. PDIA1 and β-actin were measured simultaneously in VSMC transfected with miR-mimic Ctr or 204. Measurements were performed 48 or 72 h after transfection. PDIA1 expression is recovered 72 h after transfection (n=3).

Supplementary Fig. 5 Ligated left carotid arteries were treated immediately after ligation with mimic-Ctr or miR-204 mimic (both 5 µg) and right carotid artery was maintained as control. Analyzes were performed 48 h after ligation/mimic treatment. Intimal mRNA was isolated 48 h after ligation/mimic treatment. Gene expression of VCAM-1 (A), KLF2 (B), PDIA15 (C) and CHOP (D) were performed. N=3-4. mRNA expression is normalized by its respective RCA.

Supplementary Fig. 6. Collagen-ECM were measured as described in Figure 5G. Graphs depicting high birefringent collagen (HB) in media (A) and low birefringent collagen (LB) in adventitia (B) of control RCA or 48 h after PCL (LCA) treated with miR-mimic Ctr or miR-mimic 204. N=4.

Supplementary Fig. 7. Rabbit aortic smooth muscle (RASM) cell line with conditional induction of PDIA1, were treated or not with doxycycline (dox, 1.5 µg/ml) for 24 h. After, miR-mimic Ctr or 204 were transfected. After 48 h, the expression of PDIA1 was analyzed. Dox was maintained or not during and after transfection. (A) Representative Western Blot from two independent experiments depicting Myc-tag to label exogenous PDIA1, PDIA1 and β-actin as loading control. Fluorescence quantitation is shown below each band for PDIA1 and β-actin. As expected, incubation with doxycycline promoted forced PDIA1 overexpression, which is undistinguishable from the endogenous pool, thus overriding the effects of miR-mimic 204, as discussed in text. (B) Uncropped membrane presented in "A".

Supplementary Fig. 8. Control artery (RCA) or 48 h after partial carotid ligation (LCA) in the absence (A-B) or presence of miR-204 mimic (C-D). Graphs depict delta Ct (ΔCt, Target – 18S) of PDIA1 vs. RhoGDIα from intima (A and C) or M+A (B-D). Correlation coefficient (r^2) and p value are presented in the graphs. R and L represent RCA and LCA, respectively. N=4-5 mice. Graphs A and B were previously published[10], adapted and reproduced here for clarity with permission.

Supplementary Fig. 9. Left carotid arteries (LCA) were treated with miR-mimic control (Ctr) or 204 and analysis were performed 48 h after ligation/treatment. Immunofluorescent detection of VCAM-1 (red) merged with nuclei (Dapi, blue) staining. The control contralateral artery (RCA)

of each mouse is presented above its respective LCA. Representative images from several fields from at least two independent experiments. Scale bar 10 µm.

Supplementary Fig. 10. HUVECs were submitted to laminar shear (LS) or oscillatory shear (OS) during different times (3, 6, 12, 24 or 48 h) and (A) mRNA expression of PDI (left), KLF2 (middle) and VCAM1 (right). (B) HUVECs were submitted to LS or OS during 24 h for miR-204 (left) and 211 (right) measurements. Expression values depict ratio vs. LS at each respective time point. *$p<0.05$ vs. LS; n=3-6, except for VCAM1 at 6 h time point (n=2).

Supplementary Fig. 11. HUVECs were maintained in control (Ctr) condition or treated with TNFα (3 ng/ml), after 2 h, both were washed with HBSS and full medium was replaced and maintained for additional 4 h. Graphs depict quantification of (A) mRNA expression of PDI (left), VCAM1 (middle) and CHOP (right) or (B) miR-204 (left) and miR-211 (right). Expression values depict ratio vs. Ctr. *$p<0.05$ vs. Ctr; n=3-4.
